# Supernumerary Teeth in Nepalese Children

**DOI:** 10.1155/2014/215396

**Published:** 2014-11-23

**Authors:** Varun Pratap Singh, Amita Sharma, Sonam Sharma

**Affiliations:** ^1^Department of Orthodontics, College of Dental Sciences, B.P.K.I.H.S., Dharan, Nepal; ^2^Department of Dentistry, SHKM Government Medical College, Mewat, Haryana 122107, India; ^3^Department of Pathology, SHKM Government Medical College, Mewat, Haryana 122107, India

## Abstract

*Objective*. The objectives of the present study were to investigate the prevalence and characteristics of supernumerary teeth in a patient sample of Nepalese children. *Study Design*. A survey was performed on 2684 patients (1829 females and 1035 males) ranging in age from 6 to 14 for the presence of supernumerary teeth. For each patient with supernumerary teeth the demographic variables (age and sex), number, location, eruption status, and morphology were recorded. Descriptive statistics were performed. *Results*. Supernumerary teeth were detected in 46 subjects (1.6%), of which 26 were males and 20 were females with a male : female ratio of 1.3 : 1. The most commonly found supernumerary tooth was mesiodens followed by maxillary premolars, maxillary lateral incisor, and mandibular lateral incisor. Of the 55 supernumerary teeth examined, 58.18% (*n* = 32) had conical morphology, followed by tuberculate (30.90%, *n* = 17) and supplemental (10.90%, *n* = 6) forms. The majority of the supernumerary teeth were erupted (56.36%, *n* = 31). *Conclusion*. The prevalence of supernumerary teeth in Nepalese children was found to be 1.6%, the most frequent type being mesiodens. Conical morphology was found to be the most common form of supernumerary tooth.

## 1. Introduction

Supernumerary teeth may be defined as teeth in excess of the usual configuration of twenty deciduous and thirty-two permanent teeth [1]. Their reported prevalence ranges between 0.3 and 0.8% in the primary dentition and 0.1 and 3.8% in the permanent dentition. Supernumerary tooth does not show any sexual predilection in the deciduous dentition. However, twice as many males are affected as compared to females in the permanent dentition [2–5]. The supernumerary tooth may show isolated occurrence or may be multiple, maybe unilateral or bilateral, erupted, or impacted, and can occur in either or both the jaws. Multiple supernumerary teeth are rare and are usually seen in association with cleft lip/palate, cleidocranial dysplasias, Gardner's syndrome, and so forth [6].

The exact etiology of supernumerary teeth is unknown; however, several theories have been postulated to explain their presence. The phylogenetic theory as a regression to the anthropoids whose dentition had more teeth, the autonomic recessive inheritance or linkage to the x chromosome, an abnormal reaction to a local traumatic episode, environmental factors, dichotomy of the tooth germ, and the theory of hyperactivity of the dental lamina are the most accepted ones [7].

Supernumerary teeth can be classified according to their location and morphology. The most frequent location is the maxilla, of which the mesiodens (anterior maxillary medial region) is the most commonly observed supernumerary tooth. Based on morphology, they can be classified as conical, tuberculate, supplemental, and odontomas [8, 9]. Clinically, supernumerary teeth can cause various problems locally such as retention of the primary tooth, delayed/failure of eruption of the permanent tooth, ectopic eruptions, tooth displacements, follicular/dentigerous cysts, and other alterations which require surgical or orthodontic intervention [10, 11].

The objective of the present study was to investigate the prevalence and characteristics of supernumerary teeth in the Nepalese population which is the first study of its kind in Nepal.

## 2. Materials and Methods

A survey was performed on 2864 patients (1829 females and 1035 males) ranging in age from 6 to 14 years attending the Department of Orthodontics, College of Dental Surgery, BP Koirala Institute of Health Sciences, Dharan, Nepal, over a period of two years from January 2010 to February 2012 for the presence of supernumerary teeth. This study was approved by institutional ethical review board and guidelines from the Helsinki declaration were followed. Informed consent was taken from the parents of the subjects. The patients with any syndrome or congenital anomalies such as cleft lip/palate were not included in the study. For each patient with supernumerary teeth, the demographic variables (age and sex), number, location, eruption status, and morphology of supernumerary tooth were recorded. Descriptive statistics were performed and results were tabulated.

## 3. Results

Supernumerary teeth were detected in 46 subjects (1.60%), of which 26 were males and 20 were females with male female ratio of 1.3 : 1 (Table 1).

**Figure 1:**
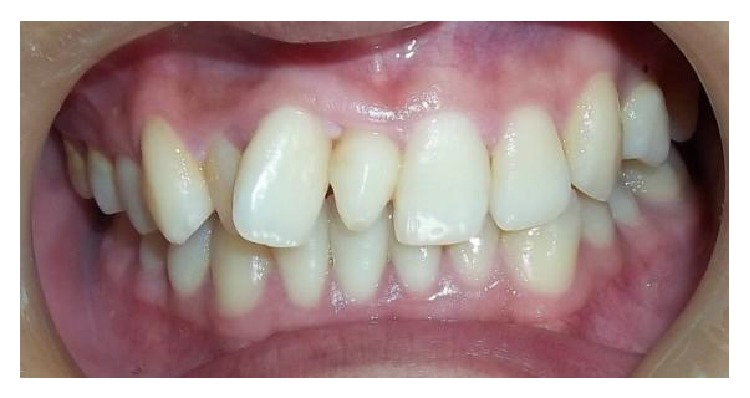
Intraoral photograph showing conical mesiodens.

**Figure 2:**
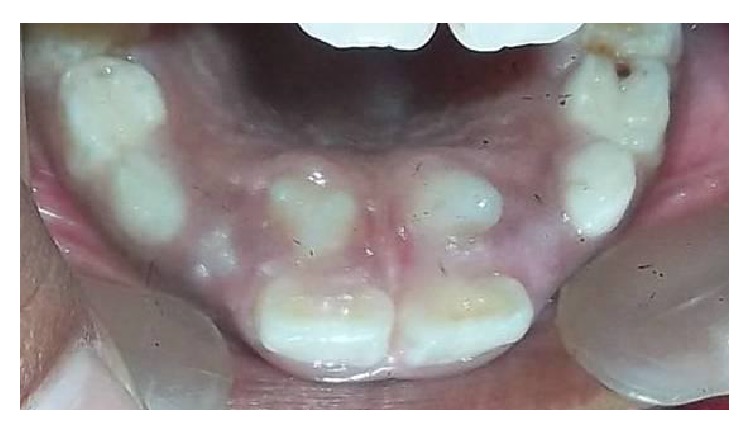
Intraoral photograph showing palatally erupted paired tuberculate supernumerary teeth.

**Figure 3:**
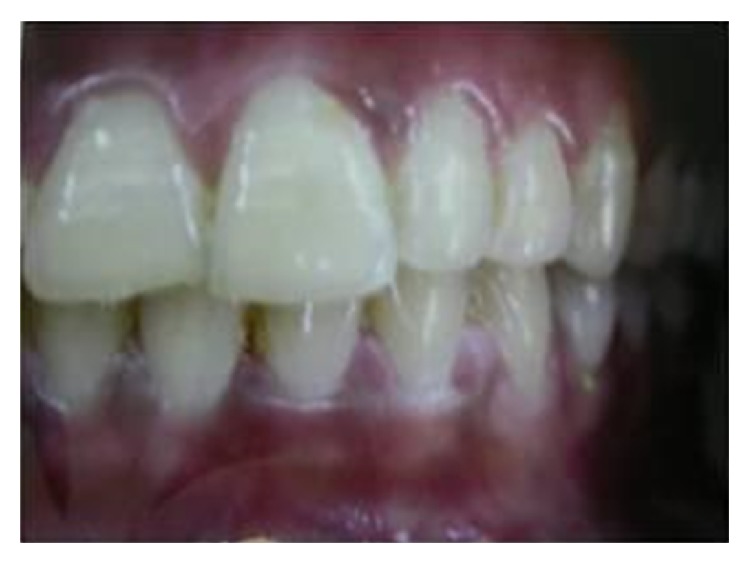
Intraoral photograph showing maxillary left supplemental lateral incisor.

Table 2 shows the characteristics of supernumerary teeth. In 82.60% (*n* = 38) of cases, one supernumerary tooth was observed, in 15.21% (*n* = 7) two supernumerary teeth were observed, and three supernumerary teeth were observed in one patient only (*n* = 1, 2.17%). A total of 55 supernumerary teeth were observed, of which 98.18% (*n* = 54) were located in the maxillary arch, while 1.81% (*n* = 1) were found in the mandible. The most commonly found supernumerary tooth was mesiodens (63.63%) followed by maxillary lateral incisor (25.45%) and maxillary first premolar (10.90%). Conical morphology was seen in 58.18% (*n* = 32) (Figure 1), while 30.90% (*n* = 17) were tuberculate (Figure 2) and 10.90% (*n* = 6) were supplemental (Figure 3) in form. Regarding their eruption status, 56.36% (*n* = 31) had erupted and 46.63% (*n* = 24) were impacted.

## 4. Discussion

Supernumerary teeth are developmental alterations that may manifest in both primary and permanent dentition, may be seen in both maxilla and mandible, and can involve any tooth. They may be associated with a syndrome or can be found in nonsyndromic patients also [21]. In our study, the prevalence of supernumerary teeth in nonsyndromic cases was found to be 1.60% and showed male predilection with a male : female ratio of 1.3 : 1.

Table 3 provides an overview of studies done on supernumerary teeth in different populations. It can be observed that the prevalence of supernumerary teeth in the Nepalese population is similar to that of Hungarian [12], Swedish [16], and Brazilian [19] population. The male : female ratio was in accordance with the studies of Gábris et al. [12] in Hungarian population, Brook [17] in British population, and Küchler et al. in Brazilian population [18]. However other studies have reported that males are affected approximately twice as frequently as females in permanent dentition [2, 13–15, 20]. These differences may be due to the differences in methodology employed and due to racial and ethnic differences in various populations [5].

In accordance with other reported studies, 82.60% (*n* = 38) of the supernumerary teeth were found to be single, 15.21% (*n* = 7) were paired, and only one case showed triple supernumerary teeth [2, 22]. Also, 98.8% (*n* = 33) of the supernumerary teeth were found to be in the maxillary arch. This value is high as compared to Salcido-García et al. [23] who found 66% of supernumerary teeth in the maxillary arch. However, it is in accordance with Simoes et al. [19] and de Oliveira Gomes et al. [11] who reported 96.7% and 91.3% of the cases in maxilla, respectively. The most commonly found supernumerary tooth has been reported to be mesiodens [19, 20, 23–25], which coincides with our findings. In order of decreasing frequency, some authors consider that mesiodens are followed by distomolars [25, 26], but others [21, 23] found that mesiodens are followed by lateral incisors and premolars. Our findings are in accordance with the latter, the mesiodens being most prevalent followed by lateral incisors and premolars. However, we were unable to find supernumerary canines and molars which may be because of the lower incidence of such teeth and further due to late developing supernumeraries [27]. In terms of morphology, conical form was most common followed by tuberculate and supplemental forms which was in agreement with the findings of other researchers [2, 11, 14, 18]. When assessing eruption status, it was found that 56.36% (*n* = 31) of the supernumerary teeth were erupted. This finding depicts a higher rate of eruption frequency than that reported by other authors [2, 11, 15, 27]. Liu [14] and de Oliveira Gomes et al. [11] showed that eumorphic teeth had a higher frequency of eruption, whereas in our study there was no significant relation between morphology of supernumerary tooth and their eruption status.

## 5. Conclusion

The prevalence of supernumerary teeth in Nepalese patients was found to be 1.60%. Males were affected more commonly than the females. The supernumerary teeth occurred more frequently in the maxilla as compared to the mandible with mesiodens being the most common type. Morphologically, conical type was the most prevalent. The majority of the supernumerary teeth were erupted.

## Figures and Tables

**Table 1:** The prevalence and distribution of supernumerary teeth in males and females.

Gender	Number	Supernumerary teeth	Frequency (%)	*X* ^2^ value	*P* value	Total (%)
Female	1829	20	1.09%	8.11	0.0043	46 (1.60%)
Male	1035	26	2.51%			

**Table 2:** Summary of the characteristics of supernumerary teeth.

Supernumerary teeth characteristics			Number (total = 55)	Percentage (%)
Number		Single	38	82.60
		Double	7	15.21
		Triple	1	2.17

Location	Maxilla (*n* = 54)	Mesiodens	35	64.81%
	98.18%	Lateral incisor	13	24.07%
		Maxillary premolar	6	11.11%
	Mandible (*n* = 1)	Lateral incisor	1	—
	1.81%			

Eruption Status		Impacted	24	43.63
		Erupted	31	56.36

Morphology		Conical	32	58.18
		Tuberculate	17	30.90
		Supplemental	6	10.90

**Table 3:** Summary of various studies carried out on supernumerary teeth in different populations.

Authors	Sample size	Country	Age	Method	Prevalence	Male : female ratio
Present study	2864	Nepal	6–14 years	Clinical examination and radiographs	1.60%	1.3 : 1
Gábris et al. (2006) [12]	2219	Hungary	15–20 years	Radiographs	1.53%	1.4 : 1
Tyrologou et al. (2005) [13]	97 children with mesiodens	Sweden	3–15 years	Clinical examination and radiographs	—	2 : 1
Rajab and Hamdan (2002) [2]	152	Jordan	5–15 years	Clinical examination and radiographs	—	2.2 : 1
Liu (1995) [14]	112 (premaxillary region)	Taiwan	4–14 years	Clinical examination and radiographs	—	2.8 : 1
von Arx (1992) [15]	90 (anterior maxilla)	Switzerland	6–10 years	Clinical examination and radiographs	—	2.6 : 1
Bodin et al. (1978) [16]	21,609	Sweden	—	—	1.6%	1.7 : 1
Brook (1974) [17]	1331	United Kingdom	11–14 years	Clinical examination and radiographs	2.1%	1.4 : 1
Yusof (1990) [3]	48,550	USA	Average 40 years	Radiographs	0.91%	—
Küchler et al. (2011) [18]	1166	Brazil	6–12 years	Clinical examination and radiographs	2.3%	1.45 : 1
Simoes et al. (2011) [19]	1719	Brazil	4–14.5 years	Radiographs	1.7%	—
Celikoglu et al. (2010) [20]	3491	Turkey	12–25 years	Radiographs	1.2%	1.8 : 1
